# Echokardiographieuntersuchungen in Zeiten der COVID-19-Pandemie: Internationale Empfehlungen und Umsetzung

**DOI:** 10.1007/s00059-020-04992-2

**Published:** 2020-10-16

**Authors:** Michael Lichtenauer, Erika Prinz, Christina Granitz, Bernhard Wernly, Apollonia Daburger, Uta C. Hoppe

**Affiliations:** Department of Internal Medicine II, Division of Cardiology, Paracelsus Medical University of Salzburg, Salzburg, Österreich

**Keywords:** Coronavirus, SARS-CoV‑2, Kardiovaskuläre Diagnostik, Infektionsrisiko, Schutzausrüstung, Coronavirus, SARS-CoV‑2, Cardiovascular diagnostics, Risk of infection, Personal protective equipment

## Abstract

Seit Dezember 2019 hat sich das neuartige Coronavirus SARS-CoV‑2 („severe acute respiratory syndrome coronavirus 2“), das die Erkrankung COVID-19 („coronavirus disease 2019“) auslöst, mit rasanter Geschwindigkeit über die gesamte Welt ausgebreitet. Ausgehend von der chinesischen Provinz Hubei, wurden mittlerweile 29,4 Mio. Menschen weltweit infiziert, und es sind mehr als 930.000 an den Folgen der Erkrankung gestorben (Stand 15.09.2020). Das Virus verbreitet sich durch Tröpfcheninfektion sowie über Aerosole. Enger Körperkontakt stellt daher ein deutlich erhöhtes Risiko für eine Übertragung dar. Insbesondere bei Untersuchungen mit engem Körperkontakt sollten daher zum Schutz nicht nur der Patienten, sondern auch des medizinischen Personals Maßnahmen ergriffen werden. In diesem Artikel werden die aktuellen Empfehlungen von internationalen Fachgesellschaften zur Verwendung von persönlicher Schutzausrüstung sowie ihre lokale Implementierung dargestellt.

## Abstract (en)

Since its first appearance in December 2019, the novel severe acute respiratory syndrome coronavirus 2 (SARS-CoV-2) has spread all over the world at a rapid pace causing the coronavirus disease 2019 (COVID-19). Originating from the Chinese province Hubei, more than 29.4 million people globally have now been confirmed to have contracted the coronavirus and more than 930,000 patients have died so far from COVID-19 (situation as of 15 September 2020). The virus is mainly spread during close contact by small droplets and aerosols. During the close contact in medical examinations, such as echocardiography, the risk of contracting the virus is increased. Therefore, the use of personal protective equipment is recommended for the protection of patients and medical personnel alike. This article summarizes the current recommendations of international societies and describes the local implementation in Austria.

Enger Kontakt zu Patienten, die an COVID-19 („coronavirus disease 2019“) erkrankt sind, stellt ein deutlich erhöhtes Risiko für die Übertragung des neuen Coronavirus SARS-CoV‑2 („severe acute respiratory syndrome coronavirus 2“) mittels Tröpfcheninfektion bzw. über Aerosole dar [1, 2]. Bei der Durchführung von TTE(transthorakale Echokardiographie)-Untersuchungen kommt es zu einem engen Patientenkontakt über einen längeren Zeitraum. Zudem könnte es insbesondere bei einer TEE(transösophageale Echokardiographie)-Untersuchung zu einer vermehrten Aerosolbildung kommen, obwohl harte Daten zur Aerosolbildung bei TEE fehlen [3]. Besonderer Schutz der Patienten und insbesondere auch des medizinischen Personals ist während dieser Untersuchungen notwendig. Die hier dargestellte Vorgehensweise beruht auf den aktuellen Guidelines der American Society of Echocardiography [4], der British Society of Echocardiography [5] sowie einer Sammlung von Erfahrungsberichten und Empfehlungen der European Society of Cardiology (ESC; [6, 7]) und den Empfehlungen des lokalen Krisenstabs an den Salzburger Landeskliniken.

Von initialer Bedeutung ist es, zwischen unterschiedlichen Kollektiven an Patienten zu unterscheiden (Patienten mit nachgewiesener COVID-19-Erkrankung, Patienten mit negativer Testung auf SARS-CoV‑2 und jene Patienten, die als möglicher Verdachtsfall definiert sind, bzw. Fälle, in denen COVID-19 noch nicht ausgeschlossen wurde), da dies einen entscheidenden Einfluss auf die Indikationsstellung zu bildgebenden Untersuchungen und Schutzmaßnahmen hat.

## Indikationsstellung

Eine strikte Indikationsstellung ist gerade in Zeiten der COVID-19-Pandemie von großer Wichtigkeit. Dies gilt sowohl für die üblichen kardiologischen Untersuchungen bei primär kardialen Patienten, die mit COVID-19 infiziert sind, als auch für Untersuchungen bei primär Herzgesunden mit COVID-19-Infektion zur Abklärung einer möglichen COVID-19-assoziierten kardialen Beteiligung [8]. Es sollten ausschließlich Untersuchungen durchgeführt werden, die eindeutig diagnostisch notwendig sind und auch eine weitere therapeutische Konsequenz nach sich ziehen würden. Dies gilt insbesondere für Patienten mit V. a. COVID-19-Infektion bzw. bereits nachgewiesener COVID-19-Infektion sowie für die Durchführung von TEE-Untersuchungen.

## Durchführung

**Figure 1:**
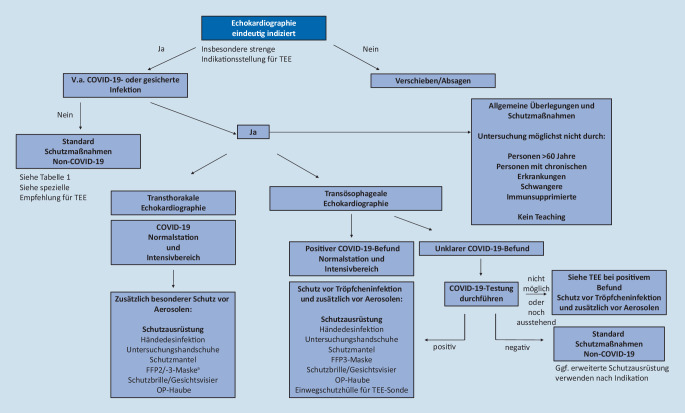

Bevor eine Echokardiographieuntersuchung in Zeiten der COVID-19-Pandemie durchgeführt wird, sollte der Infektionsstatus des Patienten nachgefragt werden, um eine Einschätzung zur Risikokonstellation treffen zu können. Je nach dem sollte danach die passende Schutzausrüstung gewählt werden (siehe Kap. 4 sowie Tab. 1 und Abb. 1). Die Durchführung einer Testung auf das Coronavirus SARS-CoV‑2 wäre gerade vor einer TEE-Untersuchung wünschenswert. Im ambulanten Setting ist dies häufig nur schwer möglich (keine Testung vorhanden oder Testergebnis noch ausstehend), in diesem Fall wäre die Verwendung einer erweiterten Schutzausrüstung empfohlen (vgl. COVID-19-Verdachtsfall, siehe Tab. 1). Patienten mit V. a. COVID-19-Infektion bzw. bereits nachgewiesener COVID-19-Infektion sollten möglichst mit einem mobilen Echokardiographiegerät untersucht werden, um eine Virusverbreitung durch Transporte zu vermeiden. Dies wäre v. a. auf speziell errichteten lokalen COVID-19-Stationen und den COVID-19-Intensivstationen empfohlen. Die Untersuchungen sollten möglichst nur von erfahrenem Personal durchgeführt werden, um die Untersuchungszeit niedrig zu halten. Untersucher im Alter über 60 Jahre, Schwangere, Personen mit Vorerkrankungen oder Immunsuppression sollten möglichst den Kontakt zu Patienten mit V. a. COVID-19 oder nachgewiesener Infektion vermeiden. Teaching oder Einschulungen am Gerät sollten nicht erfolgen. Bei V. a. COVID-19-Infektion und ausstehendem Testergebnis sollte, wenn möglich, mit der Indikationsstellung gewartet werden, außer bei dringlicher Notwendigkeit. Es sollte möglichst nur 1 Untersucher pro Raum eine Untersuchung an einem Patienten durchführen (nicht mehrere Untersucher; [6]). Empfehlenswert ist zudem, auf eine ideale Lagerung des Patienten zu achten (Patient liegt auf der linken Seite, abgewandt vom Untersucher, Verwendung eines Tuchs zum Abdecken, wenn nur Standardschutzausrüstung verwendet wird, um den Körperkontakt geringer zu halten). Die von dem Untersucher bevorzugte Schallposition sollte jedoch beibehalten werden, um nicht Abstriche bei der Untersuchungsqualität machen zu müssen oder falls ansonsten mehr Zeit benötigt werden würde. Diese Vor- und Nachteile gilt es gut gegeneinander abzuwägen. Zudem sollten, soweit möglich, direkt am Gerät nur Aufzeichnungen der Echo-Loops erfolgen sowie Messungen und Befundungen nachträglich am Computer vorgenommen werden.

**Table 1:** 

	Händedesinfektion	Handschuhe	Schutzmantel	MNS	FFP2/-3-Maske	Schutzbrille/Visier	Haube
*TTE* Standardprozedere Non-COVID-19	X	X	^a^	X	–	–	–
*TTE Normal-COVID-19-Station und Intensivbereich* V. a. COVID-19 und gesicherte Infektion	X	X	X	–	X (FFP3 im Intensivbereich)	X	X
*TEE* (positiver COVID-19-Befund, Verdachtsfall)	X	X	X	X	X (FFP3)	X	X
*TEE* (kein Verdachtsfall, aber unklarer COVID-19-Befund bzw. Testergebnis ausstehend, Testung vor TEE präferiert)	X	X	X	X	X (FFP2)	X	X
*TEE* (negativer COVID-19-Befund, je nach Begleitumständen ggf. erweiterte Schutzmaßnahmen)	X	X	Ggf.	X	Ggf.	Ggf.	Ggf.

*MNS* Mund-Nasen-Schutz, *FFP* „filtering face piece“, *TTE* transthorakale Echokardiographie, *TEE* transösophageale Echokardiographie, *COVID-19* „coronavirus disease 2019“

^a^ stattdessen Leintuch über den Patienten legen, wenn Untersucherposition rechts vom Patienten

Nach der Benützung sollten Gerät und Sonden sowie die Liege mittels Wischdesinfektion gereinigt werden. TEE-Sonden sollten wie bisher nach Herstellerangabe wiederaufbereitet werden. Des Weiteren sind die Verwendung von viruzidem Desinfektionsmittel zur Sondenaufbereitung und die Verwendung einer Schutzhülle für die TEE-Sonde aus hygienischen Gründen anzuraten.

## Verwendung von persönlicher Schutzausrüstung

Bei der Untersuchung von Patienten mit V. a. COVID-19-Infektion bzw. bereits nachgewiesener COVID-19-Infektion sollte eine umfassende Schutzausrüstung für den Untersucher verwendet werden. Empfohlen wäre hierfür nach gründlicher Händedesinfektion die Verwendung von Untersuchungshandschuhen, eines Schutzmantels, einer FFP2/3[„filtering face piece, class 2/3“]-Maske, einer Schutzbrille oder eines Gesichtsvisiers und einer Operationshaube [9]. Wir verweisen zudem auf die lokalen Empfehlungen der Krankenanstalten bezüglich COVID-19 zur korrekten Verwendung von Schutzausrüstung.

Auch bei der Untersuchung von Patienten ohne nachgewiesene COVID-19-Infektion, bei denen jedoch keine Testung erfolgt ist, wird ein erweiterter persönlicher Schutz empfohlen. Dieser umfasst nach gründlicher Händedesinfektion die Verwendung von Untersuchungshandschuhen und eines Mund-Nasen-Schutzes (MNS; siehe Tab. 1 und Abb. 1). Mittlerweile ist es außerdem eine standardmäßige Vorgabe im Arzt-Patienten-Kontakt, dass auch der Patient einen MNS trägt. Zudem wird regelmäßiges und gründliches Desinfizieren der Hände empfohlen, da dies einen essenziellen Bestandteil des Schutzes zur Prävention der Ausbreitung der Infektion darstellt.

Besondere Achtsamkeit gilt auch für TEE-Untersuchungen, da hier mit einer höheren Aerosolbildung zu rechnen ist [3]. Hierfür wäre bei positivem COVID-19-Nachweis die Verwendung von Untersuchungshandschuhen, eines Schutzmantels, einer FFP3-Maske, einer Schutzbrille oder eines Gesichtsvisiers und einer Operationshaube empfohlen. Bei unklarem COVID-19-Befund sollte möglichst vor einer TEE-Untersuchung ein Abstrich erfolgen (Ausnahme vitale Indikation). Vorzuziehen wäre generell eine COVID-19-Testung vor der Durchführung der TEE, auch um Schutzausrüstung einsparen zu können. Bei negativem COVID-19-Befund bzw. bei Patienten, die sich schon längere Zeit in Krankenhausbehandlung befanden und dabei keinerlei Symptome zeigten, kann auf die Standardschutzmaßnahmen zurückgegriffen werden. Sollten jedoch klinische oder laborchemische Zeichen einer floriden Infektion vorliegen (z. B. Abklärung Endokarditis, Fieber, C‑reaktives Protein [CRP]), wäre aufgrund der erhöhten Aerosolbelastung bei TEE-Untersuchungen und möglichen falsch-negativen Befunden eine erweiterte Schutzausrüstung zu empfehlen. Bei unklarem COVID-19-Befund und dringlicher Indikation sollte ebenfalls eine erweiterte Schutzausrüstung verwendet werden (siehe Tab. 1).

## Fazit für die Praxis

- Wegen der begrenzten Ressourcen aufgrund der SARS-CoV-2(„severe acute respiratory syndrome coronavirus 2“)-Pandemie ist eine konzise Indikationsstellung unverzichtbar.
- Wichtig zu erwähnen ist aber auch, dass keine notwendigen echokardiographischen Untersuchungen aufgrund der aktuellen Situation zum Nachteil des kardiologischen Patientenkollektivs postponiert werden.
- Bei nachgewiesenen, aber auch nicht explizit ausgeschlossenen SARS-CoV-2-Infektionen sollte zum Schutz des medizinischen Personals, aber auch anderer Patienten medizinische Schutzausrüstung während der Untersuchung verwendet werden.
